# Supplementary figures and images for: Historical record of Corallium rubrum and its changing carbon sequestration capacity: A meta-analysis from the North Western Mediterranean
Source: PLoS One. 2019 Dec 18;14(12):e0223802. doi: 10.1371/journal.pone.0223802 (PMC6919573; doi:10.1371/journal.pone.0223802)

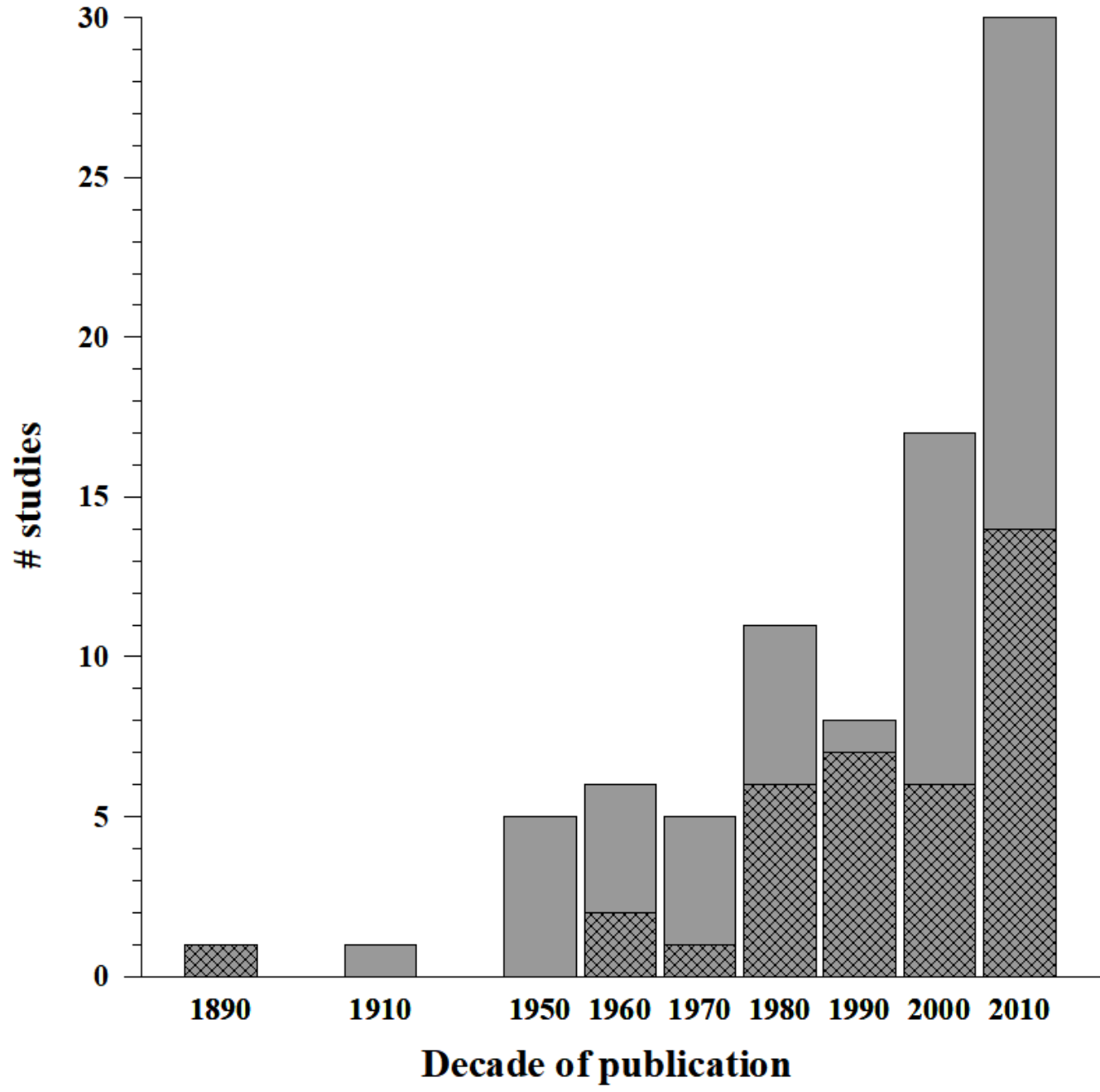

Supplement: S1 Fig — The documents that provide qualitative information represent 29% of the total. In cross grids the proportion of documents containing original data about CR basal diameter, height and/or weight: n = 37. (PDF) [file pone.0223802.s001.pdf]

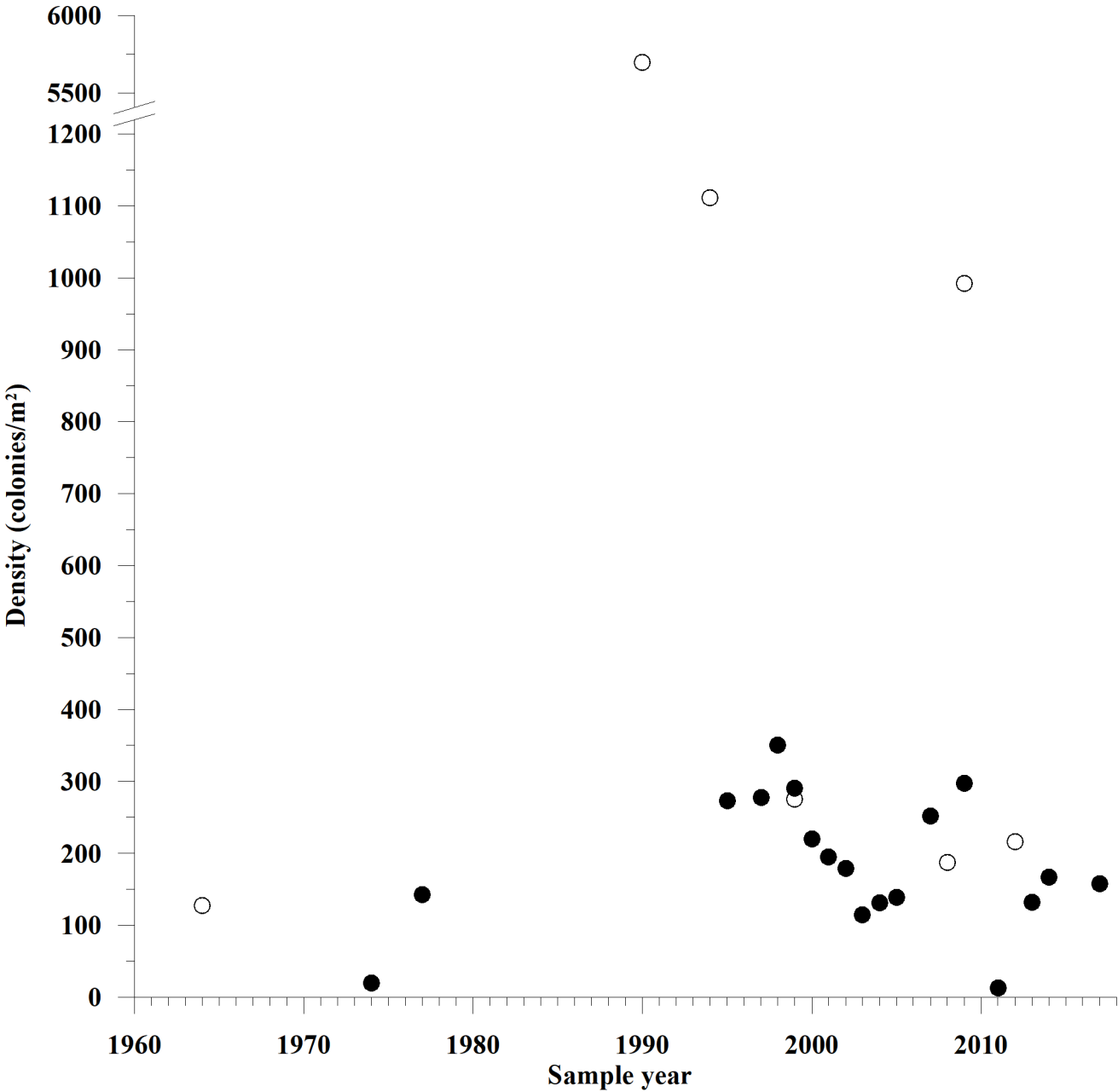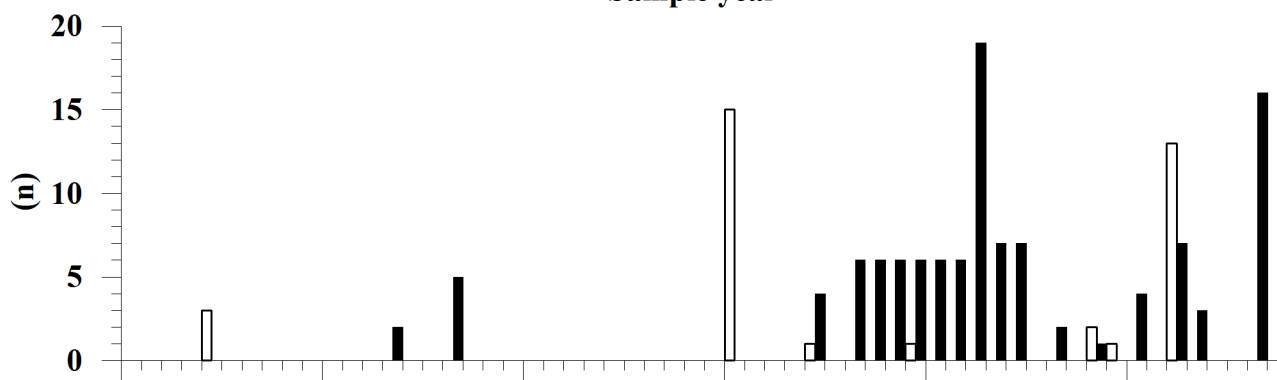

Supplement: S2 Fig — Dots correspond to the yearly average value of density. The number of data points used for the year averages are shown below. Catalan Sea: black dots and bars. Ligurian Sea: white dots and bars. (PDF) [file pone.0223802.s002.pdf]
