# Supplementary material for: Historical record of Corallium rubrum and its changing carbon sequestration capacity: A meta-analysis from the North Western Mediterranean
Source: PLoS One. 2019 Dec 18;14(12):e0223802. doi: 10.1371/journal.pone.0223802 (PMC6919573; doi:10.1371/journal.pone.0223802)
Supplement: S1 Table — The table shows the main characteristics and parameters described of the publications included in the meta-analysis, plus the publications excluded. The publications come from an extensive literature review in the three regions studied (further information on S1 Text). (PDF) [file pone.0223802.s003.pdf]

S1 Table.

Reference Code	Publication year	Class	Type	Data type	Data collection year	Region	Location	Depth range	Sampling method	Sample type	Health status parameters
**Included in the meta-analysis of this study**											
1	2018	G	Book section	QT	2017	Cat	Cap Creus, Medes & Montgrí	15-47	Photo	Random	ρ, H, B, % harvested, % necrosis
2	2015	G	Report	QT	2013	Cat	Côte Vermeille	30	Photo	Random	ρ, Nº Br, H, Ø
3	2015	A	Journal	QT	2012	Lig	Portofino	25-40	Physical	Random	ρ, H, Ø, B, W, Nº Br
4	2015	A	Conference Proceeding	QT	2012	Lig, Tus	Portofino, Tuscan Archipelago	40-230	ROV	Random	ρ, H, Ø, Occ (%), Fishing impact (%)
5	2014	A	Journal	QT	2009	Cat, Lig	Cap Creus, Portofino	30-35	Photo, Physical	Random	ρ, Rρ, Ø, A/J, Gr, Age, Fecundity, Fertility, M/F, Bored colonies (%)
6	2012	G	Report	QT	2011	Cat	Côte Vermeille	27-40	Photo	Random	H, Ø, ρ, Nº Br
7	2011	A	Journal	QT	1992-95, 1997-03, 2005	Cat	Medes & Montgrí	15-40	Photo, Physical	Random & Poached	H, Ø, ρ, Rρ, Gr, B
8	2010	A	Journal	QT	2005	Cat	Cerbère-Banyuls	23-25	Photo	Random	ρ, Ø, H, Nº Br
9	2010	A	Conference Proceeding	QT	2008	Lig	Portofino	30-70	Photo	Random	ρ, Ø, H, W, B
10	2010	A	Conference Proceeding	QT	2009*	Cat, Lig	Cap Creus, Portofino		Physical	Random	Ø, Age, Gr
11	2008	A	Journal	QT	2002, 03, 05, 06	Cat	Cap Creus, Cap Creus Canyon	50-230	ROV	Random	Patch ρ, ρ, H, Ø, Nº 1ary Br
12	2006	A	Journal	QT	2002-03	Cat	Cap Creus, Medes & Montgrí, Begur	7-49	Photo, SD	Random	ρ, Occ, Patch ρ, Ø, H, Nº Br, Age
13	2004	G	Report	QT	2004	Cat	Côte Vermeille	17-52	Photo, SD	Random, Poached	ρ, Ø, H, Nº Br, Cover (%), Necrosis, Epibiosis
14	2002	G	Report	QT	1991-95, 1997-02	Cat	Medes & Montgrí	15-40	Photo	Random	ρ, Rρ, Ø, Mortality (%)
15	2000	A	Journal	QT	1999*	Lig	Portofino	20-42	SD	Random	ρ, Ø, W, Boring (%)
16	1997	A	Book section	QT	1990, 1994	Lig	Portofino	37	Photo, Physical	Random	ρ, Ø, H, H/Apex ratio
17	1995	A	Book section	QT	1994	Lig	Portofino	58-90	ROV	Random	ρ, H, Ø
18	1993	A	Book section	QT, QL	1990	Lig	Portofino	34-42	Photo, Physical	Random	ρ, H, Ø, W, B, H/Apex ratio
19	1986	A	PhD Thesis	QT, QL	1972-74, 1977-82	Cat	Medes, Estartit	5-55	Photo, Physical	Random	H, Br Ø, Width, Organic Matter
20	1980	G	TFG	QT	1977-79	Cat	Medes	2-48	Photo, Physical, SD	Random	Vol, H, Width, Shannon Biodiversity Index
21	1979	A	Journal	QT	1978*	Cat	Banyuls	23-26	SD	Random	H, ρ
22	1965	A	Journal	QT	1964*	Lig	Portofino		Physical	Random	ρ, B
23	1963	G	Magazine	QT, QL	1962*	Lig	Genoa Bay, Portofino	20-200	Physical	Random	H
24	1895	G	Book	QT, QL	-300	Lig				Random	H
**Other studies with original health status information not included in the meta-analysis (including Tuscany)**											
25	2017	A	Journal	QT	2011, 2015	Cat	Medes & Montgrí	15-17	Photo, Physical	Poached	H, Survival rate (%), Fecundity, Fertility (%)
26	2016	A	Journal	QT	2009, 2010	Cat	La Fonera Canyon head	101-297	Echo, ROV	Random	Occ
27	2016	A	Journal	QT	2010-12	Cat	Cap Creus	25-30	Physical	Bias larger	H, Ø, Fecundity, Organic matter (%)
28	2016	A	Journal	QT	2007, 2013	Cat	Medes	15-30	Photo	Random	ρ, Cover (%), Diversity indices (coralligenous), Patch size, Patch nº
29	2016	A	Journal	QT	2008, 2009	Cat, Lig	Medes, Portofino	18, 50	Physical		Ø, Gr, Age
30	2016	A	Journal	QT		Lig, Tus	Livorno, Vada, Argentario, Portofino	30-40	Photo	Random	Cover (%)
31	2016	A	Journal	QT		Lig	Portofino	11-84	Echo, ROV, SD	Random	Cover
32	2016	A	Journal	QT	2010	Tus	Elba, Pianosa	50-130	Echo, ROV, Physical	Random	Ø, H, Age, Gr, Nº Br, Br order, Age 1st Br
33	2015	A	Conference Proceeding	QT		Cat	Medes, Cerbère-Banyuls	15-25	Photo	Random	ρ, Rρ
34	2015	A	Conference Proceeding	QT		Cat	Cerbère-Banyuls		Photo		H
35	2015	A	Conference Proceeding	QT	2012	Tus, Lig	Isuela, Montecristo, Tuna	28-108	Echo, ROV	Random	ρ
36	2015	A	PhD Thesis	QT	2002-03, 05, 06	Cat	Cap Creus	20-80	ROV	Random	Nº colonies, B, Carbon retention
37	2015	A	PhD Thesis	QT	2010-11	Cat	Cap Creus	25-30	Physical	Random	Fecundity, Gonads (nº, Ø, Vol.), Organic matter, Lipids, Fatty acids
38	2015	A	Journal	QT	2010	Tus	Elba, Pianosa	60-94	Echo, ROV, Physical	Random	Ø, H, Age, Gr, Nº Br, Age 1st Br
39	2015	A	Journal	QT	2010	Tus	Elba	85-90	Physical	Random	Ø, Fecundity, M/F, Predation (%)
40	2013	A	Journal	QT	2002	Cat	Medes	20	Physical		Gr
41	2013	A	Journal	QT	2010	Tus	Elba	50-130	Echo, ROV, Physical	Random	Occ, M/F, Fecundity, Ø, H, Br order, W, Gr, Age, ρ

42	2012	A	Journal	QT	2003-07	Tus, Cat	Calafuria, Elba, Medes	34-36	Photo, Physical	Recruits in artificial substrate	Rρ, Gr, Recruitment, Mortality, W, Epibiosis (%)
43	2012	G	MS Thesis	QL		Cat	Medes, Montgrí, Golf Pals				See S2 Table
44	2011	A	Journal	QT	2006, 2007	Cat	Medes	14-20	Photo, Physical	Random	nº species/area
45	2007	A	Journal	QT	2003, 2004	Tus, Cat	Calafuria, Elba, Medes		Photo	Recruits in artificial substrate	Mortality, Rρ
46	2007	A	Journal	QT		Tus	Livorno	25-45	Physical, Photo	Recruits in artificial substrate	Ø, Gr, Age
47	2006	A	Journal	QT	2002-03	Cat	Cap Creus, Medes & Montgrí	16-45	Physical	Random	H, M/F, Fertility, Fecundity
48	2006	A	Journal	QT	1995-96, 1997-98	Tus	Livorno	25	Photo	Random	ρ
49	2005	A	Journal	QT	1998-02	Tus	Livorno	24-36	Photo, Physical	Recruits in artificial substrate	Rρ, Ø, H, Mortality, Gr, Nº polyps
50	2004	A	Journal	QT	2002	Cat	Medes	30	Physical		Ø, Gr
51	2004	A	Journal	QT	1992, 1997	Tus	Livorno	25-40	Physical		M/F, Fecundity, Fertility, Mortality, Survival, ρ, Rρ
52	2003	A	Journal	QT		Cat	Medes	13-35	Photo	Random	Cover (%)
53	2003	A	Journal	QT	1998-99	Tus	Livorno	24-36	Physical		M/F, Fertility, Fecundity
54	2003	A	Journal	QT		Tus	Livorno	25-35	Photo	Random	Rρ
55	2003	A	Journal	QT	1998-00	Tus	Livorno	24-36	Photo	Recruits in artificial substrate	Rρ
56	2002	A	Journal	QT	1962	Cat	Cap Creus	25-35	Physical	Bias larger	Ø, H
57	1999	G	Magazine	QL		Cat	Costa Brava	5	150		See S2 Table
58	1997	A	Book section	QT		Tus	Livorno (shallow), Tuscan Archipelago (deep)	33-36 (shallow), 80 (deep)	Photo (shallow), Physical (deep)	Random (shallow); Bias larger (deep)	Shallow: Rρ, ρ, W, B; Deep: Ø, W
59	1993	A	Book section	QT	1990-91	Tus	Livorno, Elba	30-50	Physical		Ø
60	1993	A	Book section	QT		Tus	Livorno	31-36	Physical		ρ, Rρ, H, Ø, W
61	1992	A	Journal	QT	1987	Tus	Livorno	30-50	Physical	Random	ρ, Rρ, Ø, W, B
62	1989	G	Magazine	QL	1389, 1959-61, ...	Cat	Costa Brava				See S2 Table
63	1988	G	Report	QL		Lig	Portofino	16-?	Physical, SD		See S2 Table
64	1988	G	Report	QT		Tus	Livorno	31-36	Physical	Random	ρ, H, Ø, W, Recruitment (%), Dead colonies (%)
65	1988	A	Journal	QT		Tus	Livorno	31-36	Physical	Random	ρ, H, Ø, W
66	1986	A	Journal	QT	1984	Cat	Costa Brava	0-90	Physical	Bias larger	Ø, H, W
67	1986	A	Journal	QT		Cat	Cap Creus	60	Physical	Bias larger	Ø, H, W
68	1984	G	Report	QT, QL		Cat	Cap Begur to Palamós, Port de la Selva to Punta Falconera	20-50	Echo, ROV, SD, Photo, Physical	Random	B , Area, See S2 Table
69	1982	A	Journal	QL	1979, 1980	Cat	Llafranch	30-52	SD, Physical		See S2 Table
70	1978	A	Journal	QT		Cat	Banyuls	23-36	SD	Random	ρ
71	1975	A	Journal	QT, QL	1973-74	Cat	Banyuls	36	SD	Random	ρ
72	1971	G	Book section	QL	1800s-1970	Cat	Estartit, Begur, Escala, Cap Creus				See S2 Table
73	1971	A	Journal	QL	1960-61	Cat	Lacaze-Duthiers Canyon, Cap Creus Canyon	280	Physical, SDsub		See S2 Table
74	1968	A	Journal	QL	1967	Tus	Elba	26-50	SD, Echo	Random	ρ (QL), Cover (QL), Commercial value (QL)
75	1968	G	Book	QL		Lig, Tus					See S2 Table
76	1965	A	PhD Thesis	QL		Cat	Côte Vermeille	20	Physical, SD		See S2 Table
77	1961	A	Journal	QL	1959, 1960	Lig	Portofino	20-40	Physical	Random	See S2 Table
78	1959	A	Journal	QL	1959	Lig	Portofino	25-58	Physical	Random	See S2 Table
79	1958	A	Journal	QL	1955, 1957	Lig	Portofino	10-40	Physical	Random	See S2 Table
80	1958	A	Journal	QL	1958	Tus	Elba	40	Physical	Random	See S2 Table
81	1957	A	Journal	QL	1957	Lig	Portofino	20-45	Physical	Random	See S2 Table
82	1956	A	Journal	QL		Lig	Portofino		Physical	Random	See S2 Table
83	1917	G	Book	QL	1917	Cat					See S2 Table
84	Unknown	G	Generic	QL	Unknown (Within 1809-1898?)						See S2 Table
**Studies with location information (including Tuscany)**											
85	2016	A	Journal	Loc	1861-2014	Lig, Tus					
86	1985	A	Journal	Loc	1977-81	Cat	Medes		Echo, Photo, SD, Physical	Random	
87	1980	A	Journal	Loc	1972-74	Cat	Cadaqués, Estartit, Blanes, Ametlla de Mar, Sa Tuna (Cap Begur)	0-20; 0-40	Photo, Physical	Random	
88	1973	G	Report	Loc	1972-74	Cat	Cadaqués, Estartit, Blanes, Ametlla de Mar, Sa Tuna (Cap Begur)	0-45	Physical		
89	1964	G	Book	Loc		Cat	Cap Creus	15-35			
90	1962	A	Journal	Loc		Cat	Cap Creus	25-30	Physical, SD		
91	1962	A	Conference Proceeding	Loc		Lig	Portofino	10-40			
92	1916	A	Book	Loc	1916	Cat	Cadaqués, L'Escala	15-30	Physical		
93	1895	A	Journal	Loc		Cat	Banyuls, Cap Creus, Lacaze-Duthiers Canyon	25-40; >40 (Canyon)	Physical		
94	1885	G	Book	Loc		Lig, Tus	Genova, La Spezia, Tuscan Seas				

Studies with no original information (including Tuscany)

95	2017	G	Report	QT		Cat			Literature		
96	2016	A	PhD Thesis	QT	2006, 07, 13	Cat	Medes	14-30	Photo, Physical	Random	See References 28 and 44
97	2015	A	Journal	QT	1993-2012	Tus, Cat, Lig	Calafuria, Elba, Medes, La Spezia, Portofino		Physical, Photo, ROV		
98	2009	A	Conference Proceeding	QT	2008	Lig	Portofino	30-70	Photo	Random	ρ, B, W, H
99	2009	A	Journal	QT		Tus	Livorno		Literature		
100	2007	A	Journal	QT	1993-05	Cat			Literature		
101	2005	A	PhD Thesis	QT	2002-03	Cat	Cap Creus, Medes & Montgrí, Begur	7-49	Photo, SD, Physical	Random	See References 12 and 47
102	2001	G	Report	QT	1991-95, 1997-00	Cat	Medes & Montgrí	18-40	Photo	Random	ρ, Rρ, Ø
103	2000	G	Report	QT	1991-95, 1997-99	Cat	Medes & Montgrí	18-40	Photo	Random	ρ, Rρ, Ø
104	2000	G	Report	QT	2000	Cat	Medes	20-30	Photo, Physical	Random: & Poached	Ø, B + Poached: H, W, Lenght, Ø, Br Ø, Area, Vol.
105	1997	A	Journal	QT		Tus	Livorno	33-37	Photo, Physical	Random	ρ, Rρ, B, Survival
106	1995	A	Book section	QT		Tus	Livorno (shallow), Tuscan Archipelago (deep)	33-36 (shallow), 80 (deep)	Photo (shallow), Physical (deep)	Random (shallow); Bias larger (deep)	Shallow: Rρ, ρ, W, B; Deep: Ø, W
107	1995	G	Report	QT	1992-95	Cat	Medes & Montgrí	12-50	Photo	Random	Ø
108	1994	A	Journal	QT	1990	Lig	Portofino	38	Photo	Random	ρ, H, Ø, W, H/Apex ratio
109	1994	A	Journal	QT	1990	Lig	Portofino	34-42	Photo, Physical	Random	ρ, Ø, W, B
110	1982	A	Book	QT	1977-79	Cat	Medes	10-48	Physical, Photo, SD	Random	Vol, H, Width
111	1978	A	Journal	QT		Cat	Banyuls	23-26	SD	Random	ρ
112	1964	A	Journal	Loc	1960-61	Cat	Lacaze-Duthiers Canyon	280	Echo, Photo, Physical, SDsub		

See the reference of each document in the list below through its reference code.
Class: A = academic, G = grey literature.
Data type: QT = quantitative, QL = qualitative, Loc = location: a location document provides information about the presence of *C. rubrum* in a determinate place (depth and/or geographical situation) but does not give information about its health status.
Region: Cat = Catalan Sea, Lig = off Liguria, Tus = off Tuscany.
Sampling method: Photo = underwater photography and/or photogrammetry, Physical = physical samples (e.g. scraped), Echo = echosounder, ROV = Remote Operated Vehicle, SD = scuba diving observations, underwater visual census, SDsub = visual observations with submarine.
Health status parameters: Ø = basal diameter, ρ = density, Rρ = recruits density, H = height, B = biomass, W = weight, Gr = growth rate, Age: we excluded the age data obtained with the petrographic method, Nº Br = number of branches, Br order = branching order, Age 1st Br = age of first branching, Nº 1ary Br = number of primary branches, Occ = occupancy, Vol = volume, A/J = adult/juvenile ratio, M/F = male/female ratio. % = when data of the parameter is showed in relative values.
