# Supplementary material for: Historical record of Corallium rubrum and its changing carbon sequestration capacity: A meta-analysis from the North Western Mediterranean
Source: PLoS One. 2019 Dec 18;14(12):e0223802. doi: 10.1371/journal.pone.0223802 (PMC6919573; doi:10.1371/journal.pone.0223802)
Supplement: S2 Table — The information comprises notes or text extractions from the original documents. For the full reference of each code in the first column see the list of references of S1 Table. (PDF) [file pone.0223802.s004.pdf]

S2 Table.

Ref. Code	Health status information
**Catalan Sea**	
19	Shrub-like colonies, small, hard and with an irregular branching. Very variable diameter, that in the branches goes from 1 to 3 mm. Branches are located in all directions, although they tend to locate in a single level.
	Frequent species in the litoral rocky substrate, between 5 and 65 m depth. It mainly grows inside crevices and caves, where it can form facies that can surpass 100 colonies/m^2.
43	Scuba divers perceive red coral is in regression (lower density, shorter branches). Also, they perceive that high scuba diving frequency is affecting directly or indirectly the red coral.
	Perceptions of the users: Red coral had a notable regression, but it has a rising trend inside the MPA since created. The high frequency of scuba divers has a limited effect on red coral.
57	To grow 0.8 mm needs a year regarding some biologists, opinion I do not share at all, as the appreciated observations in some stretches surpass more than 1 cm.
	The red gold lives in fretworks between 5 and 150 m in semi-dark environments of caves, balms, and cliffs.
	In Catalonia, is abundant at the nord end of Girona, especially in Medes, Escala, and Cap de Creus. In Llançà is missing, only is present in a testimonial way outside Cap Ras and in Pou d'en Porret, where a coral fishing device remains from some ancestor from the area. But the little coral existant was of an excellent height and quality, not always was like that.
	Actually, scuba divers surpass the 100 m depth, as in the medium bathimetric ranges all patches are exhausted.
62	Coral growth from 6-10 m depth.
	Coral quantity per day per ship it was not usually very abundant.
	1959: Work between 30 and 40 m. The visions all the coral harvesters remember are about all the rocky walls and caves completely crowded of flowered coral with its white polips, and from the big and abundant quantity and variety of fishes and lobsters, its an inexplicable wonder, at least I can not explain it.
	Harvesting places: Illes Formigues until Sa Riera, Estartit, Medes, L'Escala (1959), Cadaqués (from punta Figuera to punta Farandell, 1961), and Massa d'Or (Cap Creus, 1966, until 95 m).
	Explained by Mr. Costas Contos: Coral was different from one area to another. The one from Begur and Cap de Creus was the best in color and without pores, but from 30 braces (~ 50 m) almost all was first quality coral, and sometimes they worked until 47 braces (~ 85 m).
68	Illes Formigues: traditional area of coral harvesting between 32-40 m. Actually present in very low densities. Sponges are observed as the main cause of polyp mortality.
	Red coral places: Ullastre, Formigues, Tamariu, Galladera, Encalladora, Portaló, Massina, Fornells, and Agullas: appearance of a bacterial veil covering the colonies.
69	Els Ullastres (Llafranch) at Winter 1979, spring and summer 1980: Closer to the 52 m depth increase the nº of colonies observed (they apear at approximately 30 m). Red coral has an habitual presence at coralligenous and semi-dark caves, in all kinds of hard subtrate inclination.
71	Port-Vendres: Found in low numbers at obscure locations.
72	High quality coral with big abundances was always bred in the submarine caves and potholes of Medes.
	Haversting places: Medes, Estartit, Escala, Cap de Creus, Begur; outside Costa Brava: Côte Bleue, Corsica, Livorno, Napoli, Genoa, Tunisia, Algeria, Morocco, until Cape Verde.
	Coral extraction almost uninterrupted since the beggining of the 1800s. In 1870-75 the first classic divers could reach 40-50 m depth. Fishing testimony in Illa Petita (Medes).
	1954: first scuba divers in Costa Brava. They accessed to the most hidden places. Harvesting magnificent coraliferous trees specimens of a massive constitution and beauty that never could be dreamt.
73	Found just at 280 m with small branches showing on the horizontal facies.
76	From about 20 m on the sciaphilic overhangs, in dense colonies.
	Common in Cap Abeille. Rare in Cap Oullestreil.
83	Predominant animals: at the beach, throwed by the waves, specially after storms, some cnidarians are found, especially corals.
84	In the countries where coral is still abundant, there are people that are dedicated to catch them, it is very easy to adquire samples from it and from other species. But it does not appear where it is not abundant or where lacks its industry, there is no other way to catch it that order the fishermen that keep the ones that casually extract sometimes with the fish, and catch the other species, that being lighter, the waves throw to the shore in the seas where they are abundant.
	The document has no date and authorship. It is believed that was done by Mariano de la Paz Graells as a guide to scientific recolection of samples by the Spanish Navy officers. M. P. Graells lived during 1809-1898 [Pérez-Rubín Feigl J. Oceanografía, biología y ordenación pesqueras en la armada española (1890-1925). In: X Congreso de la Sociedad Española de Historia de las Ciencias y de las Técnicas. Encuentro internacional Europeo-Americano 2008. Editors: Cobos Bueno JM, Pulgarín Guerrero A, Ausejo E. Badajoz, Spain. 2011;269-287.].
**Liguria**	
18	Punta Torreta: As in the earlier studies (Marchetti, 1965: Ref. 22; Tortonese, 1958; Ref. 79), we found the first red coral colonies at about 30 m.
	Punta Carega: Marchetti (Ref. 22) recorded small red coral colonies from a depth of 27 m, while Tortonese reported colonies only in the 1961 paper (Ref. 77). Today red coral starts to appear at 30 m.
	Marcante Cave: Tortonese (Ref. 79) mentioned the cave without entering into detail. Not examined by Marchetti (Ref. 22). Today there is a well-structured red coral population from the entrace to the cave reaching back 5.5 m.
	Targa: Marchetti (Ref. 22) reported the first red coral colonies at 37 m. Marchetti reported this population to be very dense, but characterized by small colonies. Also today the colonies start to appear at about 37 m, but they are less dense.
	Punta del Faro: Marchetti (Ref. 22) recorded many small colonies, starting from a depht of 21 m. Today the first colonies appear at about 25 m, and the population is characterized by a high density of small colonies.
	This study: Around the southern cliff of the Portofino Promontory the first red coral colonies, generally quite small, start to appear at about a depth of 20-25 m and they are low in density. The average density values tend to increase with depth to reach a maximum at 30-40 m.
	A comparison between our data and those of Marchetti 1965 (Ref. 22) revealed differences in the density. In 1965, this value varied from 100-300 col/m^2 with a weight between 650 and 1200 g/m^2. Therefore, in 30 years there has been an increase in density and a reduction in the mean size of single colonies. The higher density is probably due to a reduction in fishing by amateur divers, while the weight, which has remained unchanged, indicates that the increased density has led to a greater intraspecific competition. This was confirmed by the finding that colonies with the greater basal diameter occurred in stations with a lower density.
23	The colonies are usually 4 to 8 inches high (~ 10-20 cm) but may grow larger, especially in the depths.
	Red coral lives attached to rocky bottoms, between 65 and 650 feet (~ 20-200 m) along the coasts. Colonies are first found at about 65 feet but become more numerous and better deveoloped at greater depths. They are often found in remarkable numbers in the submerged and dark caves. At upper levels they are scarce and as a whole the corals are smaller and do not form dense populations. At greater depths these creatures grow on hard surfaces, forming veritable banks.
	The existence of red coral in the bay of Genoa was noticed long ago. However, this species was cosidered quite rare until 1955, when some skin divers found a number of colonies along the rocky coasts of the Portofino promontory.
24	Ligurian Sea produces branches of coral that are soft and fleshy in deep waters, while become stony when they are collected from the place where they are born. Not only its condition is modified, also the colour, as immediately they turn scarlet.
	They are branched like a tree. The branches are frequently a half foot long (15 cm); it is rare to catch branches one foot long (30 cm).
63	Location: from east of Seno de S. Fruttuoso until the head of the Portofino lighthouse.
	Coral found in vertical rock walls with overhangs and deep crevices (the closest from S. Fruttuoso Bay): red coral colonies set easily in all the crevices of the rock. Found at low depth (16m). Also, red coral is found in rocky landslides of different sizes (more frequent than the other environment).
	Marchetti (1965; Ref. 22) said red coral was absent in stations facing Cala degli Inglesi (in this study only appeared in an artificial sunken cargo boat) and the one closer to Punta del Faro di Portofino (present in this study). In this study red coral appeared with an uninterrupted distribution along all the coast studied.
	In some stations red coral appeared at shallower or deeper depths than Marchetti (1965). The stations that are found deeper now are the ones that are highly frequented by scuba divers where the collection even of small red coral had become an extended practice. The presence of many broken colonies and even partially fixed to the substrate as well as only the basis fixed to the substrate confirm this observation.
	The stations that red coral is found at shallower depths than Marchetti (1965) are the ones little frequented by divers, the approximation is difficult, the currents are strong; despite the navigation is intense vertically from the immersion areas.
	75% of colonies are parasitized, the population is massively damaged by *Clionidae* .
	Red coral colonies inside the wreck cargo boat are the biggest of the study and the colonies have the typical “bush tree” form where the ramifications develop in radius with respect to the central axis. Growth rate (at artificial cargo ship wrecked station only): 7-8 mm (new colonies, very young).
	Red coral colonies along the coast of this research are found spreading their ramification in only one dimension.
75	Its beautiful calcium carbonate skeleton has been used as jewellery, currency, and religious talismans as early as 30,000 years ago.
	First gathered in the form of fragments of natural branches thrown from the sea to the beaches. Later, in the Palaeolithic Age, a characteristic of primitive fishing, pulled away by force by divers using their hands or stones, to be then, in the Iron Age, cut with a metal device in the anfractuosities of the caves and reefs, where water was not too deep. The first fishing system is, in a certain way, confirmed by Orpheus himself, when he tells us, in narrating the already mentioned original mystery of its petrification, how coral is born far away in the sea and he dwells upon explaining to us the phenomenon of its branches swarming on the coastal gravel. The following lines would prove, furthermore, that in this sea phenomenon not only small fragments but even bigger pieces were thrown on to the beach: "Meanwhile the plant's root remain in its position, its branches bloom towards unpredictable places".
	At first, large coral branches were collected after being washed up on the beach after stroms. But soon fishermen on Greek islands started to harvest colonies from shallow water by the use of hooks on long wooden poles, and later by breath-hold diving while using Japanese goggles.
	Claudianus, an Alexandrine poet who lived at about the end of the IV Century After Christ and who, in his short poem in honour of Honorius Augustus’ marriage to Mary, spoke about the Nereid Doto, who rapidly plunging into the water pulled out corals.
	Gathering coral on the beach with an expert hand or in the maze of the sea bottoms, where the largest and most beautiful branches were picked.
	In the XIV Century, was the time when Fazio degli Uberti sang in his gentle poem: "The Ligurian Sea produces coral, at its bottom, in the shape of a small tree: the pale coloured branch between white and yellow breaks like glass, when it is caught, and the bigger it is and with more branches, the more beautiful...".
	To face the threat of impoverishment of the sea-bottoms, because of the devastating action of the devices that were being used, the Frenchs tried very hard to find better means of fishing; and the Academy of Marseilles, in 1876, announced a useless competition with a prize for the inventor of a less ravaging device.
	The favourable conditions granted by the Angevins must probably have aroused, at certain moments, the fear that coral reefs would soon be exhausted, since, by a royal edict of 1332-33, the prohibition to fish for coral without the King’s permission between Cape Minerva and Capri, a place very rich in coral, was renewed.
	In the inventory of the possessions of Guglielmo Monaco, an inventory written immediately after his death, on the 28 of September 1498, there is "a pair of paternostri of two hundred and eight big corals with a big silver gilded button all weighing one pound, one and a half ounces" (~ 500 g).
77	Punta Carega (San Fruttuoso): 20-30m: scarce and small colonies (wall), presence in crevices. At 30-40m: abundant. Numerous colonies but of small stature (walls and crevices).
78	Punta del faro di Portofino (25-58m depth): very few and small specimens. In turn, its common in all Portofino promontory.

Ref. Code	Health status information
79	Red coral locations: Punta Torretta (30-40 m): boring, competition for space. Between S. Fruttuoso and Punta Carega at 10-12 m : red coral among the most visible exponent of the fauna, at 20-40 m also present. Portofino at 20-40 m.
	In 1955, D. Marcante, during his dives, discovered the great abundance of red coral in the underwater caves near San Fruttuoso.
81	From Punta Chiappa until the East of San Fruttuoso: red coral has been emphasized by the great abundance in the caves near San Fruttuoso. The coral appears in small, low-developed colonies already at about 20 m of depth, but it is at 40-45 m that becomes dominant with taller, larger and more ramified colonies.
82	Punta Chiappa, Bay of San Fruttuoso: Substantial population of red coral, the great abundance contributes to a considerable extent (D. Marcante) although the presence of this species has been known for a long time in several localities of Liguria, including Portofino, only in 1955 Mr. D. Marcante revealed its great abundance in the caves near S. Fruttuoso.
	Deeper than 15m the coral gradually takes over and between 35-45 m becomes dominant in turn.
	Coral colonies appear already at 20-22 m in small numbers, and are characterized by a lesser stature, a weaker branching and a longer basal trunk. They can contrast with the larger, slimmer, more ramified ones that were encountered at 45m.
	Big abundance of red coral in the typical cold Mediterranean facies.
**Tuscany**	
74	Capo Fonza (Elba): red coral first apparance at 26 m with not very wide banks. Very dense banks, with very poorly developed branches, and with low commercial value.
	The branches of greatest commercial interest are those taken at greater depths by populations formed by colonies grown under these conditions.
80	Red coral at 40 m depth: covers the ceiling of small and large caves or the fissures of the coralligenous. Of excellent quality and color, although of medium thickness, the coral is surrounded by huge quantities of yellow sponges, in clusters, similar to helmets of bananas.
