# Supplementary material for: Historical record of Corallium rubrum and its changing carbon sequestration capacity: A meta-analysis from the North Western Mediterranean
Source: PLoS One. 2019 Dec 18;14(12):e0223802. doi: 10.1371/journal.pone.0223802 (PMC6919573; doi:10.1371/journal.pone.0223802)
Supplement: S4 Table — These values are used as data points for the yearly averages in Fig 4. Weight values are multiplied by a fixed density value to show biomass values in Fig 4. (PDF) [file pone.0223802.s006.pdf]

S4 Table.

Data collection year	Ref. Code	# colonies	Ø (mm)	SD	H (cm)	SD	W (g)	SD
**Catalan Sea**								
1978*	21	Unknown	9.32†	2.43	7.50		11.07ʅ	2.69
1979	20	56	6.46†	1.68	5.00		5.55ʅ	1.35
1982	19	56	6.46†	1.68	5.00		5.55ʅ	1.35
1991	14	150	2.57		1.60†	0.42		
		150	2.13					
		150	3.13		2.09†	0.54		
		150	2.21					
		150	2.98		1.96†	0.51		
		150	3.66		2.55†	0.66	1.03‡	0.48
		150	3.71		2.59†	0.68	1.13‡	0.53
1992	7	150	3.91	0.83	2.77†	0.72	1.49‡	0.70
		150	3.33	0.51	2.26†	0.59	0.44‡	0.21
		150	3.65	0.60	2.54†	0.66	1.02‡	0.48
1993		150	4.72	0.81	3.48†	0.91	2.95‡	1.38
		150	3.72	0.53	2.60†	0.68	1.15‡	0.54
		150	3.91	0.46	2.77†	0.72	1.49‡	0.70
1994		150	5.11	0.90	3.82†	0.99	3.66‡	1.71
		150	3.97	0.49	2.82†	0.73	1.60‡	0.75
		150	4.26	0.58	3.07†	0.80	2.12‡	0.99
1995		150	4.88	0.72	3.62†	0.94	3.24‡	1.52
		150	3.56	0.69	2.46†	0.64	0.86‡	0.40
		150	4.11	0.63	2.94†	0.77	1.85‡	0.87
1997		150	4.54	1.19	3.32†	0.86	2.63‡	1.23
		150	3.47	0.54	2.38†	0.62	0.69‡	0.33
		150	4.26	0.78	3.07†	0.80	2.12‡	0.99
1998		150	4.46	0.84	3.25†	0.85	2.48‡	1.16
		150	3.27	0.47	2.21†	0.57		
		150	3.85	0.57	2.71†	0.71	1.38‡	0.65
1999		150	4.14	0.96	2.97†	0.77	1.91‡	0.89
		150	3.44	0.64	2.36†	0.61	0.64‡	0.30
		150	3.67	0.62	2.56†	0.67	1.06‡	0.49
		150	7.10‡	3.33	5.78ʅ	1.40	7.26	
2000		150	4.45	0.97	3.24†	0.84	2.47‡	1.16
		150	3.28	0.58	2.22†	0.58	0.35‡	0.16
		150	3.63	0.62	2.52†	0.66	0.98‡	0.46
		133	4.62‡	2.16	3.74ʅ	0.91	2.77	
2001		150	4.52	0.93	3.3†	0.86	2.59‡	1.21
		150	3.09	0.77	2.05†	0.53		
		150	3.45	0.63	2.36†	0.62	0.66‡	0.31
2002		150	5.20	1.11	3.90†	1.01	3.82‡	1.79
		150	3.44	0.73	2.36†	0.61	0.64‡	0.30
		150	3.77	0.97	2.64†	0.69	1.24‡	0.58
2003		150	4.90	1.24	3.63†	0.95	3.28‡	1.54
		150	3.36	0.71	2.29†	0.60	0.49‡.	0.23
		150	3.58	0.82	2.48†	0.65	0.89‡	0.42
	12	1677 overall	6.55	3.16	4.61	2.90	4.68ʅ	1.14
			4.96	1.96	2.57	1.56	3.38‡	1.58
			5.73	1.98	2.65	1.57	0.37ʅ	0.09
			4.12	1.85	3.02	1.60	1.19ʅ	0.29
			4.07	1.66	2.86	1.72	0.82ʅ	0.20
			8.13	2.96	5.56	2.87	6.78ʅ	1.65
			4.71	2.20	3.30	1.62	1.81ʅ	0.44
			5.77	1.94	3.50	1.42	2.24ʅ	0.54
			4.18	1.63	2.20	1.13	1.98‡	0.93
		1722 overall	5.92	2.66	3.72	1.70	2.72ʅ	0.66
			4.09	1.31	2.49	1.34	1.82‡	0.85
			4.39	1.64	3.82	2.03	2.94ʅ	0.72
			4.12	1.66	3.55	1.84	2.34ʅ	0.57
			5.14	1.81	3.60	1.28	2.47ʅ	0.60
			4.19	1.26	2.70	1.26	0.48ʅ	0.12
			3.63	1.61	2.12	1.07	0.99‡	0.46
			4.93	1.71	3.63	1.67	2.53ʅ	0.61
			5.47	2.61	4.27	2.11	3.94ʅ	0.96
			5.10	1.88	3.75	2.16	2.80ʅ	0.68
			6.22	1.39	5.98	2.84	7.71ʅ	1.87
			4.10	1.46	2.16	1.14	1.84‡	0.86
			5.05	1.55	2.54	1.29	3.55‡	1.66
			3.76	1.14	2.41	1.21	1.22‡	0.57
			3.74	1.23	1.98	1.06	1.17‡	0.55
			4.00	1.78	2.29	1.09	1.65‡	0.77
			4.31	2.63	2.85	1.49	0.82ʅ	0.20
			4.56	2.05	3.78	1.59	2.86ʅ	0.69
2003	12	1632 overall	4.98	2.52	2.47	1.51	3.43‡	1.61
			5.20	2.05	1.97	1.36	3.82‡	1.79
			5.25	1.81	2.43	1.67	3.91‡	1.83
			4.69	1.77	2.36	1.32	2.89‡	1.35
			2.97	1.43	1.47	1.04		
		819 overall	4.75	1.72	2.52	1.45	3.02‡	1.41
			7.00	2.81	6.54	3.63	8.95ʅ	2.17
			6.44	1.97	3.59	1.02	2.44ʅ	0.59
			5.84	2.10	4.21	2.18	3.82ʅ	0.93
			5.88	2.05	4.15	2.23	3.68ʅ	0.90
			6.53	1.91	4.56	3.01	4.57ʅ	1.11
			6.75	1.95	4.00	1.68	3.34ʅ	0.81
			6.08	2.27	2.45	1.02	5.41‡	2.53
			6.45	2.79	3.02	1.86	1.18ʅ	0.29
			6.00	2.17	2.39	1.41	5.27‡	2.47
			6.01	1.96	3.91	1.41	3.16ʅ	0.77
		695 overall	4.02	1.22	2.32	1.09	1.68‡	0.79
			5.58	2.57	2.48	1.43	4.5‡	2.11
			6.43	4.32	2.07	1.24	6.06‡	2.84
			4.39	1.70	3.07	1.69	1.29ʅ	0.31
			5.58	3.71	2.82	1.44	0.73ʅ	0.18
			3.75	1.31	2.13	1.15	1.19‡	0.56
			5.13	2.17	2.29	1.33	3.7‡	1.73
		1051 overall	4.17	1.08	2.24	1.04	1.96‡	0.92
			4.23	1.42	2.36	1.15	2.06‡	0.97
			5.07	1.77	3.23	1.74	1.63ʅ	0.40
			3.95	1.45	1.61	0.98	1.56‡	0.73
2004	13	625 overall	6.00		4.80		5.11ʅ	1.24
			5.00		4.30		4.01ʅ	0.97
			6.00		4.10		3.56ʅ	0.87
			5.00		5.00		5.55ʅ	1.35
			5.00		5.75		7.2ʅ	1.75
			5.00		4.20		3.79ʅ	0.92
			6.00		5.48		6.61ʅ	1.61
			11.00		7.50		11.07ʅ	2.69
			6.00		7.00		9.96ʅ	2.42
			8.00		9.95		8.89‡	4.16
2005	7	150	4.90	1.25	3.63†	0.95	3.28‡	1.54
		150	3.36	0.70	2.29†	0.60	0.49‡	0.23
		150	3.57	0.84	2.47†	0.64	0.87‡	0.41
	8	109	5.90	3.10	6.67	3.76	9.23ʅ	2.24
2009	5	143	4.08	1.24	2.92†	0.76	1.80‡	0.84
2009*	10	36	3.67	0.78	2.56†	0.67	1.06‡	0.50
2011	2	280	5.56		3.08		1.31ʅ	0.32
		181	7.98		5.08		5.72ʅ	1.39
		251	9.01		5.69		7.07ʅ	1.72
	6	201 (Ø); 475 (H) overall	9.20	3.40	5.72	2.46	7.14ʅ	1.74
			8.60	3.70	5.64	2.69	6.96ʅ	1.69
			7.20	2.80	3.11	1.56	1.38ʅ	0.34
			5.00	1.40	2.80	1.67	0.70ʅ	0.17
2012	2	227	5.96		2.51		5.20‡	2.44
		99	5.88		3.81		2.93ʅ	0.71
		162	7.47		4.76		5.02ʅ	1.22
2013		95	5.90		2.76		0.61ʅ	0.15
		42	7.40		5.32		6.26ʅ	1.52
		78	6.00		2.20		5.27‡	2.47
		132	5.90		3.81		2.92ʅ	0.71
		31	6.70		5.77		7.25ʅ	1.76
		251	7.50		4.76		5.02ʅ	1.22
2017	1	140	5.49†	1.43	4.15	0.15	5.94	
		149	6.07†	1.58	4.66	0.20	7.49	
		109	5.44†	1.42	4.11	0.13	5.60	
		192	3.69†	0.96	2.57	0.13	2.65	
		112	5.49†	1.43	4.15	0.15	5.66	
		151	5.47†	1.42	4.13	0.13	5.66	
		110	4.51†	1.17	3.29	0.15	4.03	
		119	4.59†	1.19	3.36	0.13	3.88	
		30	7.22†	1.88	5.66	0.49	11.37	
		181	4.28†	1.11	3.09	0.25	4.28	
		130	3.86†	1.00	2.72	0.13	1.10	
		34	3.57†	0.93	2.47	0.13	4.92	
		100	3.53†	0.92	2.43	0.14	2.30	
		143	4.15†	1.08	2.98	0.17	3.81	
		52	4.24†	1.11	3.06	0.22	3.35	
		75	3.35†	0.87	2.28	0.12	1.83	
**Ligurian Sea**								
-300	24	Unknown			15.00			
1962*	23	Unknown			15.00			
1964*	22	< 100	5.64‡	2.64	4.58ʅ	1.11	4.63	
1964	3	Unknown	4.88‡	2.29	3.96ʅ	0.96	3.25	0.93
	9	< 100	4.47‡	2.09	3.62ʅ	0.88	2.50	0.40
1990	3	Unknown	2.40	0.30	3.46ʅ	0.84	2.15	0.34
	9	Unknown	5.02‡	2.35	4.07ʅ	0.99	3.50	0.40
	18	Unknown	5.40		4.07†	1.06	3.60	
			3.80		2.67†	0.70	1.50	
			4.50		3.28†	0.85	2.70	
			6.00		4.60†	1.20	2.90	
			3.40		2.32†	0.60	1.10	
			3.80		2.67†	0.70	1.40	
			5.05		3.76†	0.98	3.80	
			4.90	1.30	5.03	1.48	2.13	1.30
			5.40	1.60	5.24	1.44	2.15	1.40
1994	16	Unknown	2.50	0.90	2.38	1.23		
1999*	15	Unknown	3.50		2.41†	0.63	2.00	
2008	9	Unknown	6.46†	1.68	5.00	1.60	4.00	0.50
			12.17†	3.17	10.00	2.00		
2009	5	477	4.42	1.69	3.21†	0.84	2.41‡	1.13
2009*	10	75	4.48	1.45	3.27†	0.85	2.52‡	1.18
2012	3	368 overall	8.20	0.70	6.30	0.30	10.60	1.80
			9.10	0.90	8.60	1.30	8.50	0.90
			6.30	1.10	6.90	0.80	5.10	0.90
			7.40	1.30	6.00	0.40	6.30	1.10
			4.80	0.30	6.90	0.40	6.00	1.30
			6.60	0.80	5.80	0.60	4.70	0.70
			6.30	0.60	5.00	0.50	5.60	1.10
			5.50	0.60	5.10	0.40	5.30	1.00
			7.30	0.50	6.10	0.30	7.60	0.90
			5.90	0.30	6.20	0.43	5.20	0.50
			5.70	0.60	4.60	0.30	4.80	0.20
			4.80	0.20	4.60	0.50	3.30	0.39
	4	Unknown	7.00	2.00	7.50	5.00	11.07ʅ	2.69

See the reference of each document in the S1 Table reference list through its reference code.

colonies "overall" are stated when no # per value is available and represents the sample size containing all the values comprised until the next # shown in the table.

SD: Standard deviation.

Ø: Basal diameter. H: Height. W: Weigh.

Values obtained through formulas (See Fig. 3) are marked with signs: (†): with formula (1), (ʅ) with formula (2), and (‡) with formula (3).
