# Supplementary material for: Historical record of Corallium rubrum and its changing carbon sequestration capacity: A meta-analysis from the North Western Mediterranean
Source: PLoS One. 2019 Dec 18;14(12):e0223802. doi: 10.1371/journal.pone.0223802 (PMC6919573; doi:10.1371/journal.pone.0223802)
Supplement: S1 Text — This article was elaborated through a simplification of an original literature review. The methodology of such review is explained here, as well as the source of Fig 3, S1 and S2 Tables, plus S1 Fig. (PDF) [file pone.0223802.s007.pdf]

S1 Text.

We performed an extensive literature review about the health status of *Corallium rubrum* (CR) within the Catalan Sea (including Côte Vermeille), Ligurian Sea and off Tuscany regions. We included grey literature and qualitative documents. We did a scientific web browser research in "Topic" (Web of Science) and "In the Title" (Google Scholar) using the keywords: "Corallium rubrum" OR "red coral" OR "coralligenous" AND "Mediterranean" OR "Catalunya" OR "Catalonia" OR "Catalan" OR "Tuscany" OR "Tuscan" OR "Liguria". Additionally, we realized an in-depth methodical review of two libraries: Biblioteca Carles Bas i Peired (CSIC-CMIMA; Barcelona) and Bibliothèque du Laboratoire Arago (BUPMC; Banyuls-sur-mer); we also researched punctually in other libraries and we obtained documents from scientists. We also reviewed references inside the selected documents. We included documents written in Catalan, Spanish, English, French, Italian, or Latin. The research time span finished in September 2018.

From this search we found 102 documents describing quantitatively and/or qualitatively some parameter of CR's health status. Additionally, we found ten location documents (S1 Table). A location document provides information about the presence of CR in a definitive place (depth and/or geographical situation) but does not give information about its health status. From the original 102 documents, 84 include original information about CR's health status (S1 Fig). Among these 84 documents, we used the ones with basal diameter, height, and weight data to make Fig 3.

The documents with CR health status information (n = 84) are comprised by 80% of academic and 20% of grey literature. The documents with quantitative information represent the 79% and the qualitative ones 29% (S2 Table). The Catalan Sea is the most represented region, appearing in 52% of the articles, followed by Tuscany with 30% and Liguria with 27% (S1 Table).
