# Supplementary material for: Historical record of Corallium rubrum and its changing carbon sequestration capacity: A meta-analysis from the North Western Mediterranean
Source: PLoS One. 2019 Dec 18;14(12):e0223802. doi: 10.1371/journal.pone.0223802 (PMC6919573; doi:10.1371/journal.pone.0223802)
Supplement: Attachment — Submitted filename: CoralloPONE 2018-54.docx [file pone.0223802.s010.docx]

Manuscript Draft: PONE-D-19-15450

Historical record of *Corallium rubrum* and its changing carbon sequestration capacity: a meta-analysis from the North Western Mediterranean

The Authors described and related the *Corallium rubrum* health status in the North Western Mediterranean, in terms of morphometric variables and, potentially, as carbon sink species. A strong effort has been done to collect an important and complex data collection.

This has permitted to reach some conclusions. Obviously the different variables (basal diameter, height and / or weight for colony) were related to each other. The result more interesting is the recovery, in terms of colony size, of the protected populations after the heavy harvesting occurred in the last century. This trend, already known for some sites, today seems common to all the sites explored by the authors, suggesting that the protection measures have been effective. And this is an excellent result.

Sampling methods and statistics were performed correctly and described in detail.
In my opinion, the results are interesting, and the paper can be accepted with minor revision.
Same small remarks were done directly on the ms.
