# Supplementary material for: Historical record of Corallium rubrum and its changing carbon sequestration capacity: A meta-analysis from the North Western Mediterranean
Source: PLoS One. 2019 Dec 18;14(12):e0223802. doi: 10.1371/journal.pone.0223802 (PMC6919573; doi:10.1371/journal.pone.0223802)
Supplement: Attachment — Submitted filename: Response To Reviewers.doc [file pone.0223802.s012.doc]

**PONE-D-19-15450
Historical record of *Corallium rubrum* and its changing carbon sequestration capacity: a meta-analysis from the North Western Mediterranean: response to reviewers.**

We would like to thank reviewer R. Cattaneo-Vietti and Anonymous reviewer #2 for their useful comments and their time invested in the revision of this manuscript. We appreciate their suggestions that greatly improved the clarity of the manuscript.

Additionally, reviewer #2 raised some general remarks that we consider important. These remarks lead us to the addition of a supplementary table with data used for Fig. 4 and to deepen the discussion on the limited number of data available pre-1990s in comparison to the post-1990s.

In this document, we address each comments and issues raised by the reviewers. The reviewers’ original comments are in blue colour and our response in black. The line numbers cited in this review correspond to the ‘Revised manuscript with track changes’ document.

**Reviewer #1 - Riccardo Cattaneo-Vietti**

The Authors described and related the *Corallium rubrum* health status in the North Western Mediterranean, in terms of morphometric variables and, potentially, as carbon sink species. A strong effort has been done to collect an important and complex data collection.

This has permitted to reach some conclusions. Obviously the different variables (basal diameter, height and / or weight for colony) were related to each other. The result more interesting is the recovery, in terms of colony size, of the protected populations after the heavy harvesting occurred in the last century. This trend, already known for some sites, today seems common to all the sites explored by the authors, suggesting that the protection measures have been effective. And this is an excellent result.

Sampling methods and statistics were performed correctly and described in detail.

In my opinion, the results are interesting, and the paper can be accepted with minor revision.

Same small remarks were done directly on the ms.

L61 Comment: In my opinion, different structures, on “This community forms a complex 3D structure that serves as habitat,…”

Different literature is describing the coralligenous habitat as a complex structure in itself. E.g.: Ballesteros (2006); Perès & Picard (1964); Gerovasileiou et al. (2016). Thus, the conjunction of different microhabitats that form the coralligenous are described altogether as a single unit. For that reason, we decide to maintain the sentence as it was.

L63 Comment: animal

In these lines we are talking about the coralligenous as a key habitat, such habitat can have species of non-animal nature (Ballesteros, 2006). For this reason, we consider no modifications needed in this section of the manuscript.

L281 Comment: roman writer

Included on the new version of the manuscript. “…with the exception of the roman writer Gaius Julius…”

L381 Highlight and comment note in blank on the text: “30,000”

Unfortunately, the blank comment of the referee cannot be interpreted by us.

L456 Comment: Check better: data not confirmed, on “Cape Ampeglio [20], and La Spezia [101]”

We agree that data cannot be confirmed on Alassio and Cape Ampeglio. We eliminate those locations on the new version of the manuscript (L474-475).

Regarding Carus (1885), they mention “Spezia” as a habitat of *C. rubrum* among other places in page 62. We consider this observation valuable and report it despite its antiquity. We prefer to maintain it in the manuscript.

L462 Comment: Not for Ligurian Sea. Our data reach a max depth of 50 m, on “up to 90 m depth”.

The article we are referring in this case is Cattaneo-Vietti et al. (1995), who sampled in July 1994 with a ROV reaching 90 m in Punta del Faro as said in the original document: “*La terza immersione è stata condotta al largo di Punta del Faro, su secche affioranti circa 1-2 m dal fondo melmoso e poste, parallelamente alla costa, tra i 70 ed i 90 m di profondità.*”. We consider no modifications referring to this are needed in the manuscript.

L697: “years” instead of “Years”.

Included in the new version of the manuscript.

L721 Comment: no. He lived early 3^rd^ century AD

We acknowledge the original document was published somewhere within the 3^rd^ century BP but the published book we consulted for referencing Gaius Julius Solinus was published on 1895. We consider no modifications referring to this are needed in the manuscript.

L728 and L838: “Ministero” instead of “Ministerio”

Included in the new version of the manuscript.

L792-794 Comment: Authors?

This old document has no authorship proved. We wrote a note regarding this on the Supporting Information S2 Table. We reproduce again the note here:

“*The document has no date and authorship. It is believed that was done by Mariano de la Paz Graells as a guide to scientific recollection of samples by the Spanish Navy officers. M. P. Graells lived during 1809-1898 [Pérez-Rubín Feigl J. Oceanografía, biología y ordenación pesqueras en la armada española (1890-1925). In: X Congreso de la Sociedad Española de Historia de las Ciencias y de las Técnicas. Encuentro internacional Europeo-Americano 2008. Editors: Cobos Bueno JM, Pulgarín Guerrero A, Ausejo E. Badajoz, Spain. 2011;269-287.]*.”

L823-824 Comment: Check

Corrected and included in the new version of the manuscript as:

Torntore SJ. Italian coral beads: characterizing their value and role in global trade and cross-cultural exchange. Doctoral dissertation, University of Minnesota. 2002. p. 245.

L839-840 Comment: Check

Corrected and included in the new version of the manuscript as:

Dantan JL. Recherches sur la croissance du corail rouge (*Corallium rubrum* Lamarck). Bull soc zool Fr. 1928;53(1):42-46.

L856-857 Comment: Check

Corrected and included in the new version of the manuscript as:

Septentrion Environnement. Étude des populations de corail rouge dans la Réserve Naturelle Marine de Cerbère-Banyuls et en périphérie (Côte Vermeille). Marseille. 2015. p. 50.

L870: “marina” instead of “marine”

Included in the new version of the manuscript.

**Reviewer #2**

**General remarks:**

Mallo et al. have collated existing data on *Corallium rubrum* colony morphometric parameters from the Catalan and Ligurian Seas in order to elucidate temporal trends in these parameters, and also estimate carbon sequestration rates. This is an interesting work but there are some aspects of the methodology that need attention, or at least better justification.

Biometric parameters:

As with any work that relies on pre-existing data, a major limitation (that the authors have no control over) is the limited availability of historical data. Although the authors highlight this issue, I believe that they have not sufficiently shown whether the available data, particularly for pre-1990, is still adequate in order to make inferences on trends or otherwise. In particular, in Figure 4, the number of data points used for the year averages is exceedingly low (again, especially before 1990). For Liguria, the entire U-shaped pattern is reliant on the yearly average for 1964, which is the only pre-1990 value included and is based solely on three data points. For Catalan Sea, there are only three pre-1990 values in the graphs, each based on a single data point only. I strongly recommend that the authors: (i) explain better what a ‘data point’ is; (ii) give an indication of the number of colonies on which the yearly average data in Figure 4 is based, for each year; (iii) give due consideration to the lack of existing data when interpreting their findings, clearly indicating which of the observed patterns may be artefacts of low data availability.

We thank the reviewer for emphasizing this very important point. We have taken the following measures to address it.

(i) In the new version of the article we now clarify better what is a “data point”:

L169-172: *We used the colony patch as sampling unit for this work. When this was not possible, the nearest higher-resolution available was used. The average of all the colony patches reported parameters (or highest resolution) sampled in the same year, conformed our yearly mean value.*

L179-180: *…when samples were reportedly collected [e.g., 15, 48]. Number of colonies used to estimate each parameter can be found in S4 Table.*

L311-315, inside Fig. 4 caption: *Dots correspond to the yearly average value of the parameter. The number of data points used for the year averages are shown below each parameter graph. A data point consists in the value of the parameter from a colony patch (or the closest higher-resolution if not available).*

(ii) An indication of the number of colonies is now available in the new S4 Table (Supporting Information). S4 Table is referenced in the new version of the manuscript at L180 and L302.

(iii) We discuss that the uneven temporal distribution of historical data could bias our results (L410-415) and then we justify the use of pre-1990’s data, despite the low numbers of data points (L416-430). We consider this older information critical and they should be included to allow the comparison with recent data. The analyses of the full data set show the decreasing trend in agreement with the qualitative data gathered in this work, the FAO data (Tsounis et al. 2013), and the older commercial samples not included in the study due to collection bias towards larger colonies. In order to stress the fact that we have low pre-1990s data, we included the following text in our discussion section of the new version of the manuscript:

L50 [Abstract]: “*Quantitative data previous to the 1990s are very limited*.”

L381-383: *Furthermore, the low availability of quantitative pre-1990s data suggest caution in interpreting trends during this time period.*

L538: *Results from this work might suggest that…*

L543-544: *Such statement needs to be treated with caution due to the low availability of pre-1990s data.*

The authors do not appear to take spatial or depth variations into account in any way in their study. Could some of the differences in the yearly averages reported be simply due to the data being collected from different stations or locations?

The reviewer is right in pointing that some of the differences in the yearly average depend on the different settings of the study. This is especially noticeable in the years characterised by a low number of data points (especially pre-1990s data). Nevertheless, when aggregated, the data show a general trend that prevails above changes in the year averages due to different locations or stations sampled. Moreover, there is some spatial continuity in the dataset relative to some specific locations. In the Catalan Sea, we have studies of each main location (Cap de Creus, Medes and Montgrí, Cerbère-Banyuls) since the 2000s, showing the recent increase in CR’s biometric parameters. In the Ligurian Sea, the same location, Portofino, has been sampled since the 1960s (see S1 Table).

Regarding a possible bias due to data collected in different stations, it is unlikely to happen particularly after the 1990s, when strategically the sampling methods aimed at being representative of the entire area. Also, we only included studies that use random methods of sampling.

Moreover, in one of the analysis, not included in the manuscript, we separated the data points by sample depth (0-30 m, 30-60 m, and > 60 m). Results from this analysis showed no significant trend. We reproduce this analysis in the following graphs, where in red are shown yearly averages from colonies between 0 to 30 m depth, in pink 30 to 60 m depth, and in purple more than 60 m depth. All locations (Catalan Sea, Ligurian Sea and off Tuscany) are shown together:

The graph show that data from depths > 60 m are very scarce and only collected during the last 20 years, for this reason is not possible to draw any conclusion (as mentioned in L172-175); we only mention a hint of it (deep coral) in the discussion at L476-497. Regarding the other two depth ranges, we did not found a notable difference between both; even if there is a potential difference, the available data was not enough to make any significant conclusion. So, to increase the number of data points when calculating the yearly average we aggregated them, differentiating only between the two regions of this study.

In addition, the authors rightly highlight that recent data mostly originates from studies in MPAs and may not be representative of the situation elsewhere. Is the data for pre-1990 also from the same areas that are now MPAs?

This information can be seen in S1 Table. Concretely pre-1990 data comes from references 19 to 24 of S1 Table reference list. The locations are: Medes (two studies), Estartit, Banyuls, Portofino (two studies), Genoa Bay, and Liguria.

The study from Liguria provides no more specific location. From the seven locations remaining, five are actual MPA’s and the other two (Estartit and Genoa Bay) are located close to an actual MPA. These two locations have data in conjunction with MPA locations: one study reports data from Medes and Estartit, another from Genoa Bay and Portofino. We consider that no modifications are needed in the manuscript.

Carbon sequestration:

The statement [L216] that “The potential area where CR can live was estimated from the literature” requires further explanation. What is meant by ‘potential area’, and how was this estimated? In my opinion it is not enough to state that this was done based on data from MPAs. Is it the total area within the mentioned MPAs where *Corallium rubrum* is known to occur? Or where the habitat is considered suitable for *Corallium rubrum*, even if it is not present? Is the data from MPAs being used to extrapolate to get an estimate of ‘potential; area’ that includes the outside regions too? If so, what are the boundaries for this area estimation? From the discussion L525-531], it appears that a distinction is being made between the ‘potential area’ and ‘actual area’, with the ‘potential area’ extending beyond the MPA boundaries. This is really not obvious from the Methods section.

We thank the reviewer for raising this point, as the issue was not clearly explained in the previous version of the manuscript. In the new version of the manuscript, the potential area where CR can live is defined as the suitable surface where CR can grow, even if today’s presence is not proved or CR is not seen there (See Ballesteros, 2006). Because there are no data in the literature describing the distribution of red coral’s potential habitat for the entire Catalan Sea, we focus only on the most studied locations. Such locations are the three Catalan MPAs, and coincide where almost all the Catalan CR is found: Cap de Creus, Medes Islands and Montgrí coast, and Cerbère-Banyuls. For these locations we searched three reference articles where the hectares of CR’s suitable substrate were available:

(i) Sardà et al. (2012): Cap de Creus. Platform coralligenous and coralligenous with Paramuricea: 116.8 ha. (ii) Hereu and Quintana (2013): Medes i Montgrí. Coralligenous with gogonians, conglomerate rocky coastlands colonized mainly by animals, dark and semi-dark caves and tunnels: 0.136 ha. (iii) Payrot et al. (2014): Coralligenous: 56.4 ha.

Then, to be consistent, we use the same approach for the Ligurian Sea. The most studied sites where almost all the CR is found is the Portofino MPA, which has a suitable substrate for CR of 23.8 ha regarding Cánovas-Molina et al. (2016). In summary, our “potential area” is defined as the suitable surface where CR could grow, considering the areas where most of the CR has been recorded (coinciding with this four MPA’s).

No extrapolations to other areas where done in our results section. The area for those calculations is limited to the 23.8 ha of Liguria and the 173.3 ha of the Catalan Sea. In the discussion section we decide to use further data from Cánovas-Molina et al. (2016). In their study they calculate 23.8 ha of suitable substrate for CR’s growth, but also identify 6.31 ha, within the 23.8 ha, of actual red coral facies (“actual area”). Additionally, they cover the entire Ligurian Sea coastal region identifying 130.9 ha of suitable substrate for CR growth (potential area but this time not only considering the areas where most CR can be found; this is an extrapolation considering areas outside the MPA area). Those two surfaces are used in the discussion section to compare the present situation what we have today vs. the potential situation inwhat we could have in the entire Ligurian Sea.

A similar procedure was made for Coppari et al. (forthcoming), considering the suitable substrate and making extrapolations. See also Coppari (2015).

To better clarify this section that has caused confusion we added the following changes on the revised manuscript:

L224-232: *The potential area where CR can live was estimated from the literature. We define “potential area” as the surface suitable for CR growth even if CR is not currently present in the area. In both regions, we limited the extension of the potential areas to the locations where most CR is found. For the Catalan Sea we used data from the three marine protected areas (MPAs): Cap de Creus [55], Medes i Montgrí [56], and Cerbère-Banyuls [57]. From the Ligurian Sea we used the data from Portofino (MPA since 1998) by Cánovas-Molina et al. (2016) [58].*

L547-555: *An example illustrates the importance of CR distribution for carbon storage: the potential area where CR can grow in the entire Ligurian Sea accounts for 130.9 ha, whereas the actual CR specimens have an extension of 6.31 ha, only found in Portofino MPA [58]. This would account for a C flux of 0.0009 kg C ha-1 y-1 in 2012, notably smaller than the value extrapolated from the entire Ligurian Sea’s potential area (0.0190 kg C ha-1 y-1).*

I consider the use of a fixed density value for estimating total coral biomass [L208-215] as problematic for two reasons. First, the very existence of widely varying density data (S2 Fig), while possibly due to different methodologies, is also likely a result of actual spatial variation in coral density (e.g. Scandola has higher *Corallium rubrum* densities than elsewhere, as highlighted in L531-537). If density varies from site to site, then using a fixed density value may lead to an inaccurate estimate of total carbon sequestration. More problematic, however, is that this fixed density value is being combined with the colony size measurements to estimate carbon sequestration rates. But with density fixed, the carbon sequestration rate becomes a function of colony size. Yet Cau et al. (2016) have strongly indicated that *Corallium rubrum* populations are influenced by self-thinning processes, where population density and mean colony size are inversely related. Therefore, it is not appropriate to assume that density is fixed and that only colony size is changing.

We agree with the referee about possible CR density variability, in addition to bias relative to the different methodologies used, density of red coral populations has a spatial and temporal variability. In this study we did not aim to reach this detail and complexity as we are limited by our data sources (scarce data from the literature and use of different methodologies). However, we are making the best possible approximation for the estimate of the CR overall biomass in the selected sites and the carbon sequestration study: we use a rough conservative estimate using the mean colony size values and the most representative density value of the shallow colonies in each of both regions.

We consider such approximation the most reasonable, similar approaches than our are also realized in studies like Chambers et al. (2001), Connor et al. (2001), Chmura et al. (2003), Duarte et al. (2010), Marbà et al. (2014), Krause-Jensen and Duarte (2016), Pan et al (2011), Coppari (2005), and Coppari et al. (Sci. Rep.; forthcoming).

We add the following text in order to highlight our conservative approach and the implications of using a fixed density in our calculations:

L221-224: *The use of a mean fixed density value involves an estimate of the CR biomass for the selected sites, as we do not take into account the spatial and temporal variability of CR’s density [30, 32].*

L244-246: *…use a relation of 5.82 mg cm-1 to convert the increase of growth to weight. Like we did for the biometric parameters, we use the same fixed density values, not taking into account the CR’s self-thinning processes [30]. To estimate the potential for…*

L516-521: *…where practically all the data comes from (Cap de Creus, Medes & Montgrí, Cebère-Banyuls, and Portofino). Furthermore, we assumed a fixed density value despite density could change in space (location and depth), time, and due to its relation with the colony size (self-thinning process) [30, 32]. According to our calculations, since the 1990s until today, the potential carbon…*

Ref. 30 = Cau et al. (2016), ref. 32 = Linares et al. (2011).

**Other general remarks:**

The PLOS Data policy requires authors to make all data underlying the findings described in their manuscript fully available without restriction… For example, in addition to summary statistics, the data points behind means, medians and variance measures should be available.

The authors have only partly addressed this requirement, because the actual data used to calculate the yearly average values shown in Fig. 4 is not provided anywhere. The authors should include these data as supplementary material, either as part of S1 Table or as an additional supplementary table.

The data used to construct the average values of Fig. 4 can be found in the S4 Table of the Supporting Material of the new article version.

**Specific comments:**

L200-207: Higher r² statistically means that most of the variation in ‘Y’ is explained by ‘X,’ and therefore that knowing the ‘X’ value one should be able to get a good prediction on the ‘Y’ value. It is therefore very strange to see that Eqn 3, which had the highest r² value, is indicated as having the greatest prediction error, while Eqn 2, which had the lowest r², is shown as having the smallest error.

We revised the r^2^ and % of error formulas and procedure and we found no mistake. We attach together with the “Response to reviewers” document an excel file with the calculations of the % error named “Response to reviewers attachment 1”. We consider that no modifications are needed in the manuscript.

L420-423: The authors are attributing changes in biometric parameters from the 1990s onward to the ban of trawling and *ingegno* fishing. But my impression is that colonies found at the depths covered by the present work, i.e. down to 60m, were especially targeted by Scuba diving, whereas trawling and *ingegn*o fishery was used more at deeper depths.

We have no scientific evidence of which harvesting system was more used on this decade and the immediate previous years. Our logic says that both systems were used: the non-specific trawling or dredging plus the use of the *ingegno* or the St. Andrew’s cross and the specific red coral scuba diving harvesting. The former was able to cover wider areas in less time, thus being more profitable in economic terms on the areas where red coral was abundant. The latter picked the coral found in more hidden places where the other system cannot reach.

Trawling or dredging was also realized on shallower seafloors, as this technique of fishing is effective on a more or less horizontal surface, which can be found up 60 m in some coastal areas. Proofs of that fact can be found in other habitats (i.e. seagrasses):

Tserpes et al. (2006): “*Intense anthropogenic activities, prevailing along the Mediterranean coast, have devastating effects on the environment and, particularly, on seagrass beds, which are considered by international conventions to be endangered habitats.* Posidonia oceanica *seabeds, in particular, are experiencing a widespread decline throughout the Mediterranean Sea. In order to reduce the negative effects of fishing on such habitats, the EU has banned trawling within three miles of the coast or in depths of less than 50 m (whatever comes first).*”

De Juan et al. (2007) and Demestre et al. (2008): These articles study a trawled fishing ground on a continental shelf off the Ebro Delta between 30 and 80 m depth.

González-Correa et al. (2005): This article study *P. oceanica* meadows affected by trawling in Alicante between 14 and 28 m depth.

As we did not found any specific scientific proof of a shallow coralligenous habitat of our study area exploited by these fishing techniques, we decided to remove the part mentioning *ingegno* and trawling as an explanatory hypothesis of our results in the new version of the manuscript:

L55-59 [Abstract mentioning trawling is now modified]: “*The halt in the C. rubrum decreasing trend coincided with the status of overexploitation in the existing populations and the first recovery response due to protection in some areas, suggesting that these protection measures…*”

The following text on L438-442 is now deleted: “*Finally, the decrease of CR parameters values in the 1990s was likely due to the ban of trawling and the use of ingegno in Spain implemented in 1986, and in the entire Mediterranean in 1994 [85; Council Regulation No. 1626/94]*”.

Several other minor comments or suggestions are included in an annotated version of the manuscript.

L27 Comment: Reword: …research on the health status of Corallium rubrum, …

Included in the new version of the manuscript.

L29 Comment: Population what? Population density/abundance? Popultion size-structure? Population parameters?

Changed in the new version of the manuscript as: “To reconstruct the temporal trends of the most reported *C. rubrum* population parameters in the Northwestern…”

L31 Comment: The changes are not in the species but in its rate of carbon sequestration; reword: …to determine changes in total carbon sequestration by this species.

Included in the new version of the manuscript.

L73 Comment: Which phyla are being referred to here? Just anthozoans?

We refer here to benthic suspension feeders that retain carbon. Apart of anthozoans we might find also bryozoans, polychaetes, sponges… We consider that no modifications are needed in the manuscript.

L74 Comment: “for which those structures lose part of their function as carbon sinks”: Meaning not clear.

Sentence deleted in the new version of the manuscript as the meaning of it was a repetition in other words of the previous sentence.

L122 Comment: CR is a species, it cannot be a biodiversity hotspot… it’s role is as a habitat-forming species/ecosystem engineer.

Changed in the new version of the manuscript as: “…including CR’s role as a carbon sink or as a habitat-forming species”

L135 Comment: “anatomy”: Not sure what this is supposed to mean in the context of a population

We decided to delete the sentence between commas in order to avoid confusion. Now remains like that in the new version of the manuscript: “…describe the characteristics of a population and serve to determine the…”

L202 Comment: Not clear if this is based on the same data that were used to construct the regression lines, or completely different data

It is based on the same data used for the construction of the regression lines. In order to clarify, it is changed in the new version of the manuscript as: “with experimental values obtained from the previous literature research.”

L205 Comment: The regression equation with the highest r2 value was Eqn 3. Higher r2 statistically means that most of the variation in ‘Y’ is explained by ‘X,’ and therefore that knowing the ‘X’ value one should be able to get a good prediction on the ‘Y’ value.

It is therefore very strange to see that here Eqn 3 is indicated as having the greatest prediction error, while Eqn 2, which has the lowest r2, is shown as having the smallest error.

Please check.

See comment above at page 16, inside Referee #2 specific comments.

L215 Comment: So you are assuming that CR density did not change at all during the entire time period covered by the study, and that only size changed?

But density and size can be inversely related, see Cau et al. (2016).

See comment above at page 13, inside Referee #2 general remarks.

L216 Comment: How? What criteria were used? What do you even mean by “potential area”?

See comment above at page 11 inside Referee #2 general remarks.

L229-231 Comment: What do these values show? Basal diameter or colony height increase per year?

Changed in the new version of the manuscript as: “The CR’s basal diameter growth rate was obtained…”.

L420-421 Comment: Increase? L413: In the 1990s the decreasing trend stopped…

We assume that the reviewer meant “Decrease?” instead of “Increase?” in his/her comment. We thank the reviewer for noticing our slip. Changed in the new version of the manuscript as: “Finally, the stop in the decrease of CR parameters..”.

L421-422 Comment: My impression is that colonies at the depths covered by the present work, i.e. down to 60m, were especially targeted by Scuba diving, whereas trawling and ingegno fishery was used more at deeper depths.

See comment above at page 17, inside Referee #2 specific comments.

Grammatical corrections:

Grammatical corrections proposed by the reviewer on L65, L66, L69, L82, L106, L122, L147, L407, and L463 were accepted by the authors and included in the new version of the manuscript.

The grammatical correction proposed by the reviewer on L176 was not accepted by the authors and thereby not included in the new version of the manuscript. The referee proposed substitute “quadrants” by “quadrat”, but we maintain “quadrants” as it is the name of the delimited areas used to do a methodological approach to measure red coral’s parameters. E.g.: Drap et al. (2014), Weinberg (1979), Montero-Serra et al. (2015).

Reference:

Cau et al (2016) Habitat constraints and self-thinning shape Mediterranean red coral deep population structure: implications for conservation practice. Scientific Reports, 6:23322. DOI: 10.1038/srep23322

Once again, we thank you for helping us to improve the manuscript. In the eventuality of a publication, we would like to acknowledge your contribution explicitly.

**Attachments:**

Excel document: “Response to reviewers attachment 1”

**References:**

Ballesteros E. Mediterranean Coralligenous Assemblages: a Synthesis of Present Knowledge. Oceanogr Mar Biol An Annu Rev. 2006;44:123–95.

Cánovas-Molina A, Montefalcone M, Vassallo P, Morri C, Bianchi CN, Bavestrello G. Combining literature review , acoustic mapping and in situ observations: an overview of coralligenous assemblages in Liguria (NW Mediterranean Sea). Sci Mar. 2016;80(1):7–16.

Carus JV. Prodromus Faunae Mediterraneae Vol. I. Koch E, editor. Stuttgart; 1885.

Cattaneo-Vietti R, Bavestrello G, Cerrano C. Prime osservazioni sul corallo rosso profondo lungo il promontorio di Portofino. In: Seconda ricerca sul corallo rosso nelle acque italiane. Centro Lubrense Esplorazioni Marine: Ministero per le risorse agricole, alimentari e forestali; 1995. p. 104–12.

Cau A, Bramanti L, Cannas R, Follesa MC, Angiolillo M, Canese S, et al. Habitat constraints and self-thinning shape Mediterranean red coral deep population structure: implications for conservation practice. Sci Rep. 2016;6:1–10.

Chambers JQ, Higuchi N, Tribuzy ES, Trumbore SE. Carbon sink for a century. Nature. 2001;410:429–429.

Chmura GL, Anisfeld SC, Cahoon DR, Lynch JC. Global carbon sequestration in tidal, saline wetland soils. Global Biogeochem Cycles. 2003;17(4):12.

Connor RF, Chmura GL, Beecher CB. Carbon accumulation in Bay of Fundy salt marshes: implications for restoration of reclaimed marshes. Global Biogeochem Cycles. 2001;15(4):943–54.

Coppari M, Zanella C, Rossi S. The importance of coastal gorgonians in the blue carbon budget. Sci Rep. Forthcoming 2019.

Coppari M. The importance of benthic suspension feeders in the biogeochemical cycles: active and passive suspension feeders in coralligeneos community. Universitat Autònoma de Barcelona. Universitat Autònoma de Barcelona; 2015. p. 119.

Demestre M, Juan S De, Sartor P, Ligas A. Seasonal closures as a measure of trawling effort control in two Mediterranean trawling grounds: Effects on epibenthic communities. Mar Pollut Bull. 2008;56:1765–73.

Drap P, Merad D, Mahiddine A, Seinturier J, Gerenton P, Peloso D, et al. In situ underwater measurements of red coral: Non-intrusive approach based on coded targets and photogrammetry. Int J Herit Digit Era. 2014;3(1):123–39.

Duarte CM, Marbà N, Gacia E, Fourqurean JW, Beggins J, Barrón C, et al. Seagrass community metabolism: Assessing the carbon sink capacity of seagrass meadows. Global Biogeochem Cycles. 2010;24(4):1–8.

Garrabou J, Sala E, Linares C, Ledoux JB, Montero-Serra I, Dominici JM, et al. Re-shifting the ecological baseline for the overexploited Mediterranean red coral. Sci Rep. 2017;7:0–6.

Gerovasileiou V, Dailianis T, Panteri E, Michalakis N, Gatti G, Sini M, et al. CIGESMED for divers: Establishing a citizen science initiative for the mapping and monitoring of coralligenous assemblages in the Mediterranean Sea. Biodivers Data J. 2016;4:1–23.

González-Correa JM, Bayle JT, Sánchez-Lizaso JL, Valle C, Sánchez-Jerez P, Ruiz JM. Recovery of deep Posidonia oceanica meadows degraded by trawling. J Exp Mar Bio Ecol. 2005;320:65–76.

Hereu B, Quinana X. El fons marí de les illes Medes i el Montgrí: quatre dècades de recerca per a la conservació. Hereu B, Quinana X, editors. Càtedra d’ecosistemes litorals Mediterranis; 2013. 195 p.

Juan S De, Thrush SF, Demestre M. Functional changes as indicators of trawling disturbance on a benthic community located in a fishing ground (NW Mediterranean Sea). Mar Ecol Prog Ser. 2007;334:117–29.

Julius Solinus G. Collectanea rerum memorabilum. Berolini, editor. 1895. p. 392.

Krause-Jensen D, Duarte CM. Substantial role of macroalgae in marine carbon sequestration. Nat Geosci. 2016;9:737–42.

Linares C, Garrabou J, Hereu B, Diaz D, Marschal C, Sala E, et al. Assessing the Effectiveness of Marine Reserves on Unsustainably Harvested Long-Lived Sessile Invertebrates. Conserv Biol. 2011;26(1):88–96.

Marbà N, Díaz-Almela E, Duarte CM. Mediterranean seagrass (Posidonia oceanica) loss between 1842 and 2009. Biol Conserv. 2014;176:183–90.

Mazzarelli G. Banchi di corallo esplorati dalla R. Nave “Volta” nell’estate del 1913. Roma: Ministerio di agricoltura, industria e commercio; 1915. p. 178.

Montero-Serra I, Linares C, García M, Pancaldi F, Frleta-Valic M, Ledoux JB, et al. Harvesting effects, recovery mechanisms, and management strategies for a long-lived and structural precious coral. PLoS One. 2015;1–14.

Pan Y, Richard A, Fang Y, Houghton R, Pekka E, Werner A, et al. A large and persistent carbon sink in the World’s forests. Science (80- ). 2011;333:988–93.

Payrot J, Hartmann V, Cadène F. Plan de gestion 2015-2019 de la Reserve Naturelle Marine de Cerbère-Banyuls, section A, diagnostic de la RNMCB: informations générales, environnement, biodiversité, cadre socio-économique et culturel, vocation à accueillir le public et intérët pédagogique. Conseil Général des Pyrénées-Orientales. 2014. p. 85.

Peres JM, Picard J. Noveau manuel de bionomie benthique de la Mer Mediterranee. 1964. 137 p.

Sardá R, Rossi S, Martí X, Gili JM. Marine benthic cartography of the Cap de Creus (NE Catalan Coast, Mediterranean Sea). Sci Mar. 2012;76(March):159–71.

Tserpes G, Peristeraki P, Nielsen JR. Ecological Side-effects of fishing from the fisheries management perspective. In: The Knowledge Base for Fisheries Management. Developments in Aquaculture and Fisheries Science; 2006. p. 267–94.

Tsounis G, Rossi S, Bramanti L, Santangelo G. Management hurdles for sustainable harvesting of Corallium rubrum. Mar Policy. 2013;39:361-364.

Weinberg S. The light-dependent behaviour of planula larvae of Eunicella singularis and Corallium rubrum and its implication for octocorallian ecology. Bijdr tot Dierkd. 1979;49(1):16–30.
