# Supplementary material for: Historical record of Corallium rubrum and its changing carbon sequestration capacity: A meta-analysis from the North Western Mediterranean
Source: PLoS One. 2019 Dec 18;14(12):e0223802. doi: 10.1371/journal.pone.0223802 (PMC6919573; doi:10.1371/journal.pone.0223802)
Supplement: Attachment — Submitted filename: Response To Reviewers.doc [file pone.0223802.s013.doc]

**PONE-D-19-15450R1
Response to reviewers, round 2.**

**Historical record of *Corallium rubrum* and its changing carbon sequestration capacity: a meta-analysis from the North Western Mediterranean.**

We would like to thank reviewer #1 for examining the revised manuscript and supporting its publication and the reviewer Julian Evans for providing further comments.

In this document, we address each reviewer’s comment. The reviewer’s original comments are in blue and our response in black.

Finally, we would also like to acknowledge your contribution as editor and reviewers.

**Reviewer #1**

The manuscript describe a technically sound piece of scientific research with data that supports the conclusions. The conclusions are drawn appropriately based on the data presented. I suggest the publication, being the revision suggestions accepted.

We kindly thank the reviewer for the time invested in the revision process that greatly improved the manuscript.

**Reviewer #2 – Julian Evans**

I have read with interest the revised version of the manuscript by Mallo et al. on historical records of Corallium rubrum and consequent changes its carbon sequestration rate, as well as their detailed response letter to the reviewers’ feedback.

I consider that the authors have adequately addressed my previous comments and the manuscript is now nearly acceptable for publication, but recommend the following minor amendments:

L53-55: “The halt in the C. rubrum decreasing trend coincided with the status of overexploitation in the existing populations” >> The meaning of this phrase is not fully clear to me… Perhaps: “The halt in the C. rubrum decreasing trend coincided with overexploitation of the existing populations”?

In order to gain clarity, we decided to update the conclusions it in the following way:

“The halt in the *C. rubrum* negative impact coincided with the exhaustion of tree-like colonies and the first recovery response due to effective protection measures in some areas. Nevertheless …”.

We decided to do in this way because we think the reviewer’s suggestion could also be confusing, as this halt tendency occurred when such populations where mostly found already overexploited, and not when this overexploitation was going on.

L38: “of” should not be in italics

Included in the new version of the manuscript.

L73: “Moreover, compared to larger colonies, the younger (and smaller) colonies possibly retain less carbon in their structures” >> In their reply, the authors have indicated that “We refer here to benthic suspension feeders that retain carbon. Apart of anthozoans we might find also bryozoans, polychaetes, sponges…” Since polychaetes and sponges are not colonial, the term “colonies” may not be the best choice of word, so I suggest they consider alternative wording.

We appreciate the reviewer’s comment here. We consequently modify the text in the new version of the manuscript, which now reads as follows:

“Moreover, compared to larger specimens, the younger (and smaller) ones possibly retain less carbon in their structures”.

L146: “antiguity” should be “antiquity”

Included in the new version of the manuscript.

L165: It would be ideal to briefly what “colony patch” means…

In the new version of the manuscript we now include a definition of colony patch:

“We used the colony patch as sampling unit for this work. We consider a patch of red coral as a distinct group of colonies, where the distance of colonies within the patch is substantially lower than the distance of colonies between patches (CR usually follows a patchy distribution) [14]. When the colony patch sampling unit was not possible, the nearest higher-resolution…”.

[14] = Rossi et al. 2008.

L166-168: “The average of all the colony patches reported parameters (or highest resolution) sampled in the same year, conformed our yearly mean value.” >> I suggest rewording this sentence to: “For a given parameter, the yearly mean value was taken as the average of the values for all the colony patches (or higher resolution) sampled in the same year.”

Included in the new version of the manuscript as worded by the referee.

L226: “From the Ligurian Sea” should be “For the Ligurian Sea”

Changed in the new version of the manuscript.

L508: “Furthermore, we assumed a fixed density value despite density could change in space (location and depth), time, and due to its relation with the colony size (self-thinning process)” is better written “Furthermore, we assumed a fixed density value despite the fact that density can change in space (location and depth), time, and due to its relation with colony size (self-thinning process)”

Changed in the new version of the manuscript as worded by the referee.

**References:**

Rossi S, Tsounis G, Orejas C, Padrón T, Gili JM, Bramanti L, et al. Survey of deep-dwelling red coral (Corallium rubrum) populations at Cap de Creus (NW Mediterranean). Mar Biol. 2008;154:533-545.
